# A Mobile Serious Game About the Pandemic (COVID-19 - Did You Know?): Design and Evaluation Study

**DOI:** 10.2196/25226

**Published:** 2020-12-22

**Authors:** Juliano De Souza Gaspar, Eura Martins Lage, Fernando José Da Silva, Érico Mineiro, Isaias José Ramos De Oliveira, Igor Oliveira, Rayner Guilherme De Souza, Juan Rodrigues Oliveira Gusmão, Camila Fernanda Donadoni De Souza, Zilma Silveira Nogueira Reis

**Affiliations:** 1 Faculdade de Medicina Universidade Federal de Minas Gerais Belo Horizonte Brazil; 2 Departamento de Tecnologia de Design, Arquitetura e Urbanismo Universidade Federal de Minas Gerais Belo Horizonte Brazil; 3 Curso de Design Universidade Federal de Minas Gerais Belo Horizonte Brazil

**Keywords:** coronavirus, COVID-19, e-learning, mHealth, digital health, gamification, serious game, mobile apps, public health, informatics

## Abstract

**Background:**

No treatment for COVID-19 is yet available; therefore, providing access to information about SARS-CoV-2, the transmission route of the virus, and ways to prevent the spread of infection is a critical sanitary measure worldwide. Serious games have advantages in the dissemination of reliable information during the pandemic; they can provide qualified content while offering interactivity to the user, and they have great reach over the internet.

**Objective:**

This study aimed to develop a serious game with the purpose of providing science-based information on the prevention of COVID-19 and personal care during the pandemic while assessing players’ knowledge about COVID-19–related topics.

**Methods:**

The study was conducted with the interdisciplinary collaboration of specialists in health sciences, computing, and design at the Federal University of Minas Gerais, Brazil. The health recommendations were grouped into six thematic blocks, presented in a quiz format. The software languages were based on the progressive web app development methodology with the Ionic framework, JavaScript, HTML5, cascading style sheets, and TypeScript (Angular). Open data reports of how users interact with the serious game were obtained using the Google Analytics application programming interface. The visual identity, logo, infographics, and icons were carefully developed by considering a selection of colors, typography, sounds, and images that are suitable for young audiences. Cards with cartoon characters were introduced at the end of each thematic topic to interact with the player, reinforcing their correct answers or alerting them to the need to learn more about the disease. The players’ performance was assessed by the rate of incorrect and correct answers and analyzed by linear correlation coefficient over 7 weeks. The agile SCRUM development methodology enabled quick and daily interactions of developers through a webchat and sequential team meetings.

**Results:**

The game “COVID-19–Did You Know?” was made available for free on a public university website on April 1, 2020. The game had been accessed 17,571 times as of September 2020. Dissemination actions such as reports on social media and television showed a temporal correspondence with the access number. The players’ error rate in the topic “Mask” showed a negative trend (*r*=–.83; *P*=.01) over the weeks of follow-up. The other topics showed no significant trend over the weeks.

**Conclusions:**

The gamification strategy for health education content on the theme of COVID-19 reached a young audience, which is considered to be a priority in the strategy of orientation toward social distancing. Specific educational reinforcement measures were proposed and implemented based on the players’ performance. The improvement in the users’ performance on the topic about the use of masks may reflect an increase in information about or adherence to mask use over time.

## Introduction

### Background

Two months after the first reports of COVID-19 in China, the first case was officially registered in Brazil [1,2]. At this time, the new coronavirus (SARS-CoV-2) had already been identified in more than 50 countries, accounting for 87,000 cases and 3000 confirmed deaths according to the World Health Organization (WHO) [3]. In November 2020, on the date of preparation of this paper, more than 52.4 million cases of COVID-19 and 1.2 million deaths due to the disease had already been registered worldwide [3]. In the same period, in Brazil, more than 5.8 million cases and 164,000 deaths by COVID-19 were registered [4].

In the absence of a short-term treatment or vaccine, providing access to information about SARS-CoV-2, the transmission route of the virus, and ways to prevent the spread of infection has been the focus of health strategies. To help countries prepare themselves to face the pandemic, the WHO has provided guidance and training on how to prevent and delay the transmission of the disease. Personal hygiene recommendations, such as washing hands frequently with soap and water, wearing a mask in public, avoiding handshakes, and social distancing whenever possible, continue to be widely disseminated in the population through various communication channels [5]. However, for such measures to be effective, in addition to government boosting, community awareness and engagement are considered to be critical factors to control disease [6].

The disease caused by SARS-CoV-2 is relatively mild in young adults, teenagers, and children [7]. Most people in these age groups, even when infected, are asymptomatic or oligosymptomatic; this creates concern regarding the potential of this population to transmit the disease, especially by direct contact with people in high-risk groups [8]. Also, physical and social distancing drastically reduced the opportunities for collective engagement among young people, causing psychological distress in many and leading to breaking of the distancing pacts that are so relevant at this time [9]. Another important consideration is the ease with which adolescents can use technology, which is vital to keep communication channels open and help adolescents inform and support each other in addition to sharing information with most of the community.

Digital health solutions can be a promising approach to address the spread of COVID-19; digital tools can effectively support institutions, facilitating the wide dissemination of information [10]. Communication during a pandemic must reach the target population in a timely fashion and provide clear, objective information. Combating misinformation and fake news about the origin, dissemination, and treatment of COVID-19 is a strategy that enables citizens to increase their adherence to the measures recommended during the crisis [11]. Thus, a good communication strategy avoids confusion and distrust, which can have negative consequences for individuals and society [11].

In the context of strict rules on social distancing, a serious digital game can offer significant advantages for the dissemination of information and learning because it does not require the user’s physical presence, increases their interactivity with the content, and provides wide information coverage [12]. A serious game is characterized as a game in which the main purpose is not entertainment and fun [13,14]. Serious games can be powerful tools for the development and acquisition of new knowledge and skills by experienced users as well as by beginners [15].

### Objective

The aim of this study was to develop and evaluate a serious game for mobile platforms to provide information about prevention of COVID-19 and personal care during the pandemic.

## Methods

### Study Design

The applied research in this study has an interdisciplinary profile between medicine, computer science, and design. In this study, the development, implementation, and evaluation of a serious game that addresses topics related to preventive measures and information about COVID-19 is presented. Teenagers are the target audience for this serious game; however, it can also be played by literate children and adults.

### Theoretical Basis

A literature review on COVID-19, gamification, serious games, mobile apps, and e-learning was conducted. Concerning the topics on COVID-19 and prevention recommendations, it was decided to use only the information and recommendations available on the WHO website [3]. In addition to the initial research, periodic queries were made to the WHO website to obtain updates on recommendations and guidelines for action. A team of specialists, including physicians, professors, and medical students, reviewed and validated all content used in the serious game.

### Learning Objectives

The learning content of this serious game was grouped into six topics that presented specific WHO recommendations for the population, with an emphasis on issues related to the daily lives of teenagers:

- Coronavirus: information about COVID-19 and vulnerable groups
- Mask: why and how to wear masks
- Take Care: transmission of COVID-19
- Cleaning: care for cleaning the home and tools for work and study
- Health: personal health, routines, and life habits
- Social: socializing with friends and school, how to shop, care outside the home

### System Requirements

Based on the learning objectives and the identification of the target population, the system requirements were defined and a search of specialized websites on the characteristics of frequently used devices was conducted. Items such as screen resolution and the amount of internet access via mobile phone, tablet, or desktop [16], as well as aspects such as reach, necessary network specifications, and data consumption were analyzed. The design team also considered specific characteristics for gamification and gameplay [15]. The most important requirements that guided the development are listed below.

- The game is accessible on different platforms (mobile and desktop web).
- It does not require registration to use.
- It does not use gaming platforms that require robust hardware, that is, it is possible to run it with simple processors and little memory.
- It consumes little internet browsing data.
- It maintains the player’s score history,
- It enables sharing of the results of the phases on social networks.
- It enables users to view their personal and global ranking in the game.
- It offers information complementary to the questions of the game.
- It offers information about the project, the institution, and the team.

### Design and Development

Agile SCRUM development methodology [17] was used in this project, and a multidisciplinary team was systematically gathered around the project. Weekly tasks were defined for each participant according to their qualifications. The tasks were developed during a cycle (“sprint”) lasting 1 week [18]; rapid daily interactions were conducted through a webchat and weekly meetings with the entire team (on the web) to present the results for the week and define new tasks for the new sprint.

To create a multiplatform application, the progressive web app (PWA) software development methodology with the Ionic framework, using JavaScript, HTML5, cascading style sheets (CSS), and TypeScript (Angular) programming languages, was used. For the development, the Visual Studio Code tool [19] was used, and the code management was controlled with the Bitbucket tool [20] to enable the team to collaborate on codes and test them through the Git versioning system [21]. The Web Storage application programming interface (API) [22] was used to store information in the user's browser about the use and performance of the questions.

Open data reporting from the Google Analytics API was used to obtain information on how users interact with serious gaming [23]. Only information related to the use of a game is collected, such as the type of device, screen resolutions, clicks on right and wrong answers, how users accessed the game (via external links, social networks, search engine searches, news sites), and the time spent in the game. Even considering only very generic data, data retention by Google Analytics was configured for maximum storage of 26 months on its servers [24].

### Communication and Diffusion of the Game

Complementary actions were implemented to diffuse the game. Among these, the creation of social network accounts on Facebook [25] and Instagram [26] are the most notable, in addition to sending emails to the population previously registered for the Faculty of Medicine of Universidade Federal de Minas Gerais (UFMG) newsletter [27]. Emails were also sent specifically to coordinators and teachers of elementary and local high schools.

### Statistical Analysis

The data used in this analysis were retrieved from the Google Analytics weekly reports. Data such as the access number and the numbers of right and wrong answers were analyzed.

Continuous variables are represented as median values and categorical variables for absolute and relative values. Specifically, for the numbers of right and wrong answers, we calculated the hit rates and error rates for each topic aggregated by week. The Pearson correlation coefficient was used to assess the temporal variation over the weeks, and the Student *t* test was calculated to assess the significance of the results found by the coefficient. The significance used was *P*<0.05, and the calculations were performed using Google Sheets.

Line graphs were used to present the variation of error rates over the weeks evaluated, and the trend line was presented with the Pearson linear correlation coefficient (*r*) for topics with statistical significance. Sector charts and maps were also used to describe the categorical variables.

### Ethical Aspects

This project does not use sensitive user data because the developed game does not involve user registration and does not have an associated database. The data used in the analysis of this study were generated by indirect reports (generated by Google Analytics) and with temporary data regarding access to the site. This project seeks to comply with all data usage laws in force in Brazil and Europe, including clarifying the indirect use of nonsensitive data to the user in an objective, clear, and timely manner. The project was developed and supported by the Center for Health Informatics of the Faculty of Medicine and the School of Architecture at UFMG.

## Results

The game “COVID-19–Did You Know?” was made available for free on the web on April 1, 2020. Using the PWA methodology, the game was published on the server of the Faculty of Medicine of UFMG; submission to app stores was not required, and the game could be accessed directly on the website [28].

During the development process with the SCRUM methodology, 23 cycles lasting one week were performed. In each cycle, several independent tasks were assigned to the team members, including the literature review, definition of system requirements, quiz preparation, development of the game logic, interfaces, player ranking, final design, software testing, English and Spanish translations, and publication of the game on the web.

### Learning Objectives

**Figure 1:**
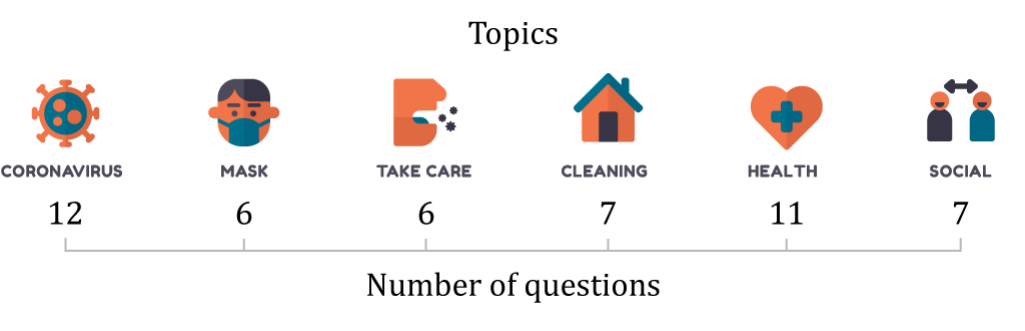
Learning objective topics and number of questions for each topic.

Learning objectives were defined based on the target population, and the information was grouped into the topics Coronavirus, Mask, Take Care, Cleaning, Health, and Social. Figure 1 shows the six topics with the number of questions created in each set.

### The Game

**Figure 2:**
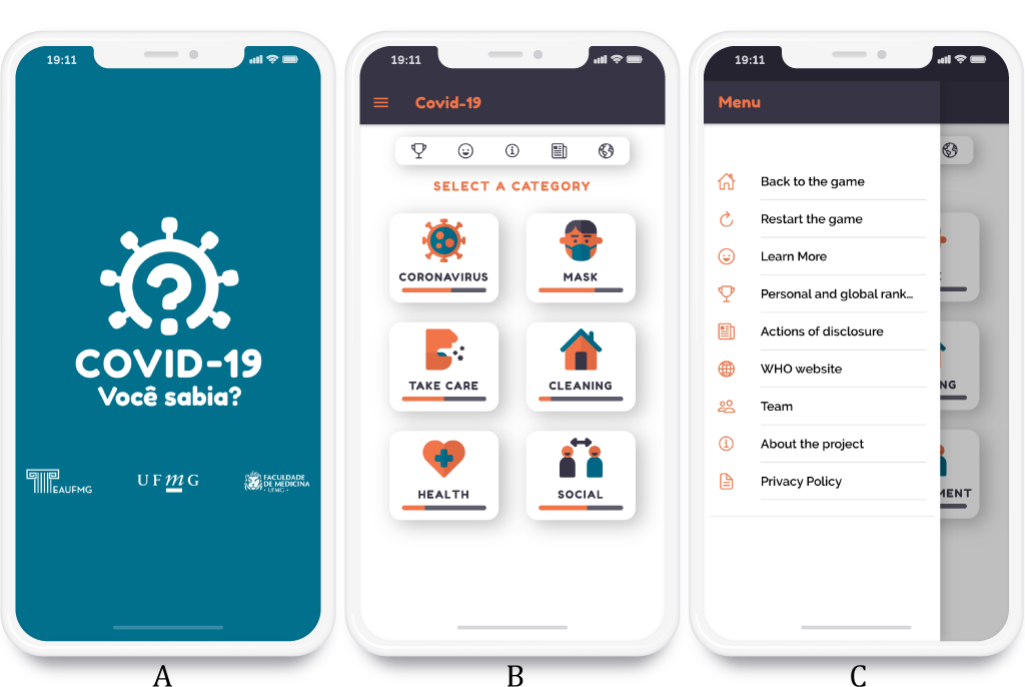
Interfaces of the initial screens of the game: Splash (A), Home (B), and Menu (C).

Design experts created the visual identity of the app. A dedicated logo and icons were developed (Figure 1); also, colors and typography were selected to meet the requirements defined in this project. The Attribution-Non-Commercial-Share-Equal 3.0 Brazil license [29] allows the free use of all images, infographics, and information pieces created for noncommercial purposes. Figure 2A shows the splash screen interface, which uses a minimalist concept with the game logo and the brands of the School of Architecture and the Faculty of Medicine of UFMG.

The main interface of the serious game, shown in Figure 2B, has buttons to access each set of questions on the defined topics. Along with each icon, a progress bar is displayed showing the player's evolution in the topic. A quick access bar was created at the top, providing the player with easy access to their ranking, changing the language of the game, and other information. In the upper left corner, a standard menu button gives access to all the resources and information in the game (Figure 2C). In the menu, it is possible to reset the score of the game, access the WHO website, learn more information about the topics, and learn more about the project and development team, in addition to consulting the complete Privacy Policy.

The game was developed in multiple languages to reach an audience beyond the borders of Portuguese-speaking countries. The second version of the app became available in September 2020; in addition to the Portuguese language, it added English and Spanish language options. In this version, all questions, answers, feedback, and infographics, as well as the Privacy Policy and complementary information, were translated into the new languages.

**Figure 3:**
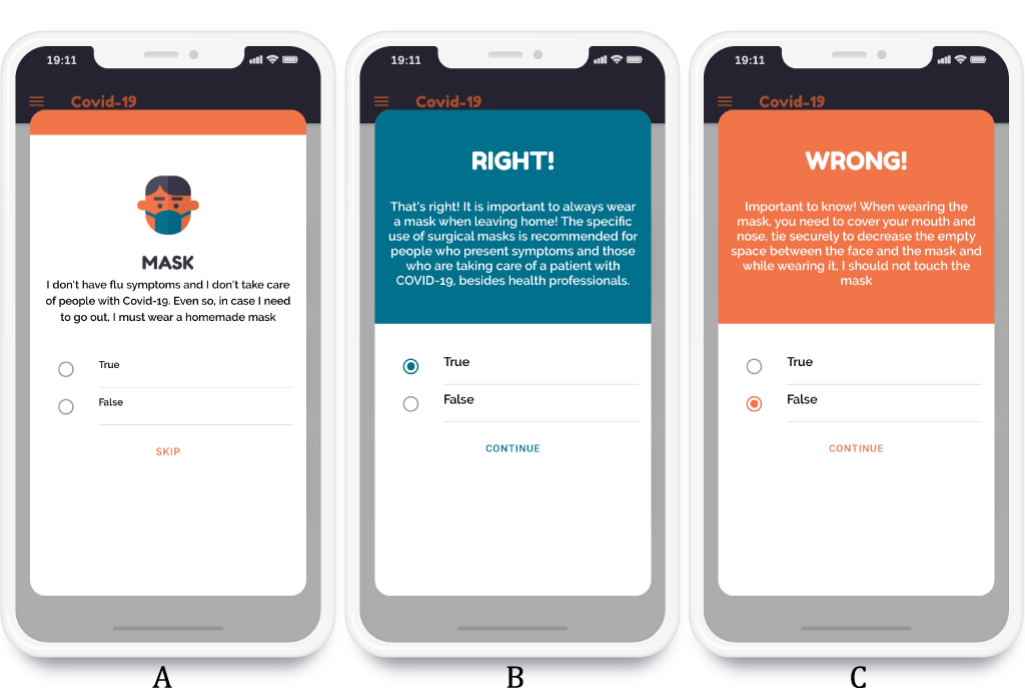
Interface showing a question (A), feedback for the right answer (B), and feedback for the wrong answer (C).

When the player selects a topic in the main interface (Figure 2B), they are presented with a set of questions; they can then answer or skip each question (Figure 3A). When the player answers the question, feedback about their right or wrong answer is presented to them (Figure 3B, 3C). Audiovisual effects, including icons, colors, and sounds, were used for the questions as well as for each type of feedback. All images have the alt attributes of the HTML5 language defined according to World Wide Web Consortium recommendations [30]; these attributes help to describe an image when it cannot be reproduced by the browser or even when the user employs reading aid software for the visually impaired.

**Figure 4:**
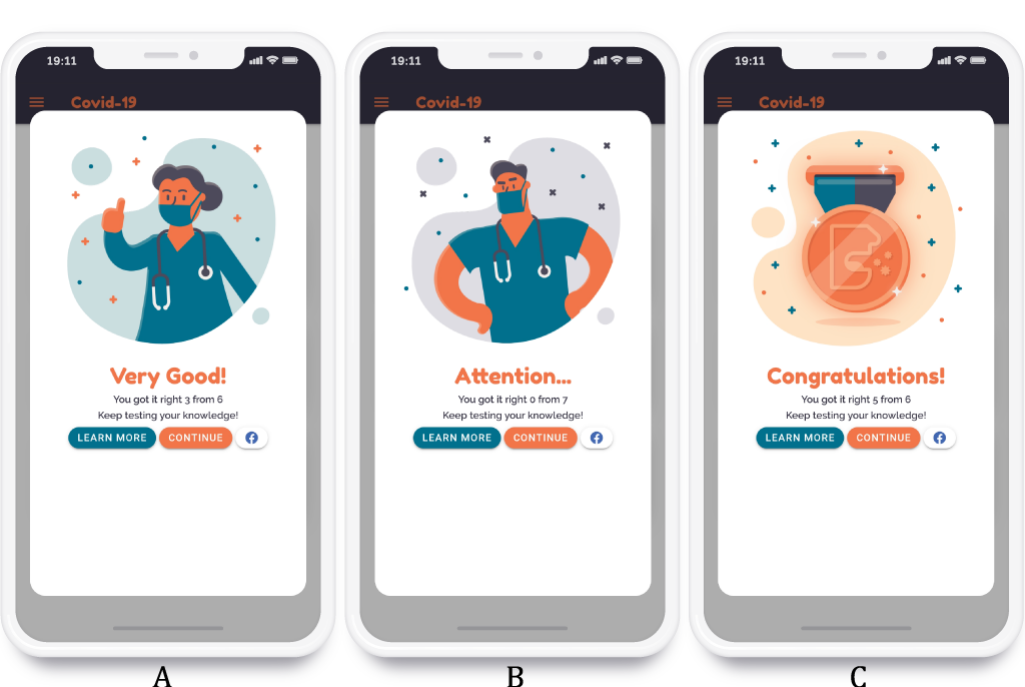
Cards with results (A, B) and medal (C) for a topic.

When the player finishes a topic, a card with a cartoon of a physician appears, congratulating the player for answering the questions and finishing that topic (Figure 4A) or asking them to pay more attention to the topic (Figure 4B). Female and male versions of the cartoon physician appear randomly. The user is also awarded different medals for each successfully completed topic (Figure 4C). The card also contains buttons the user can click to learn more about the topic, continue to the next topic, or post their performance on Facebook.

**Figure 5:**
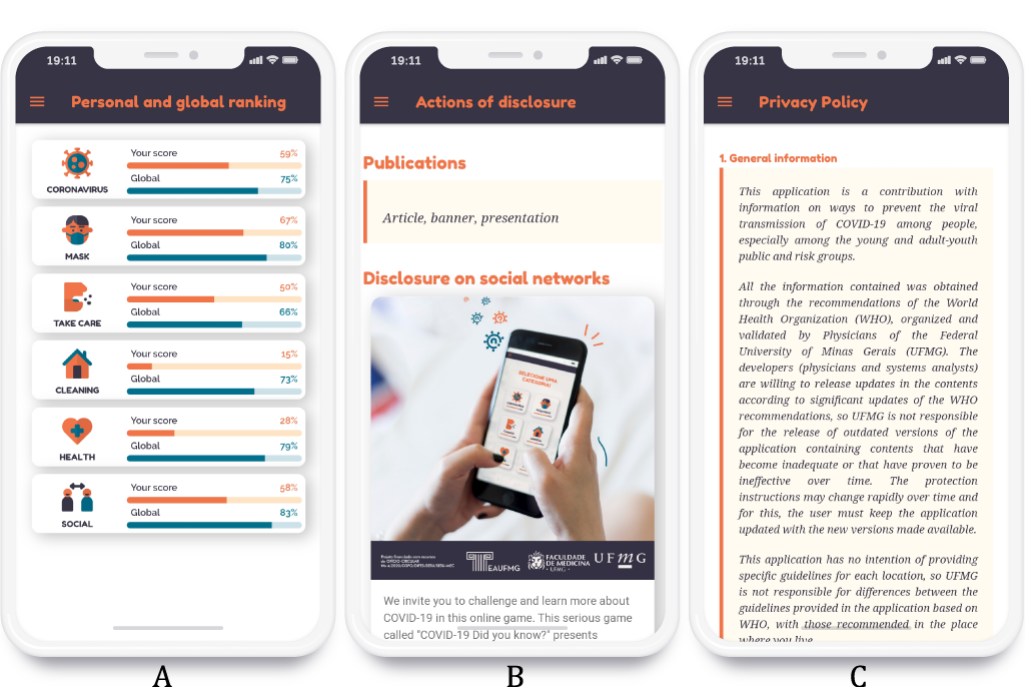
Personal and global ranking (A), disclosure actions (B), and Privacy Policy (C) screens.

The Local Storage resource was employed to save user data locally, and Google Analytics was employed to collect nonsensitive user data. To comply with the General Data Protection Regulation (GDPR) in force in Europe and the corresponding Brazilian legislation, a notice about the use of the data is presented as soon as the app starts. This notice informs the user that no personal data are collected but that “cookies” and similar methods can be used to save their score [31]. The use of nonsensitive data is also requested, and the user is invited to read and accept the Privacy Policy containing detailed information (Figure 5-C). The complete text of the Privacy Policy [32] is provided in Multimedia Appendix 1.

The player's score is stored in their browser using Web Storage technology; therefore, it is possible to show a progress bar on the main screen indicating the user’s evolution by topic (Figure 2B). This also enables the user to compare their performance with that of other players in the global ranking interface (Figure 5A). Moreover, we implemented an algorithm so that when the user selects a topic they have already played, only questions they have not yet answered or for which they gave wrong answers are shown. The global ranking of right answers by topic was calculated from the reports generated by Google Analytics, which in this case collects the number of user clicks on correct or incorrect answers. Users can also opt to disseminate images from the game on social networks (Figure 5B).

**Figure 6:**
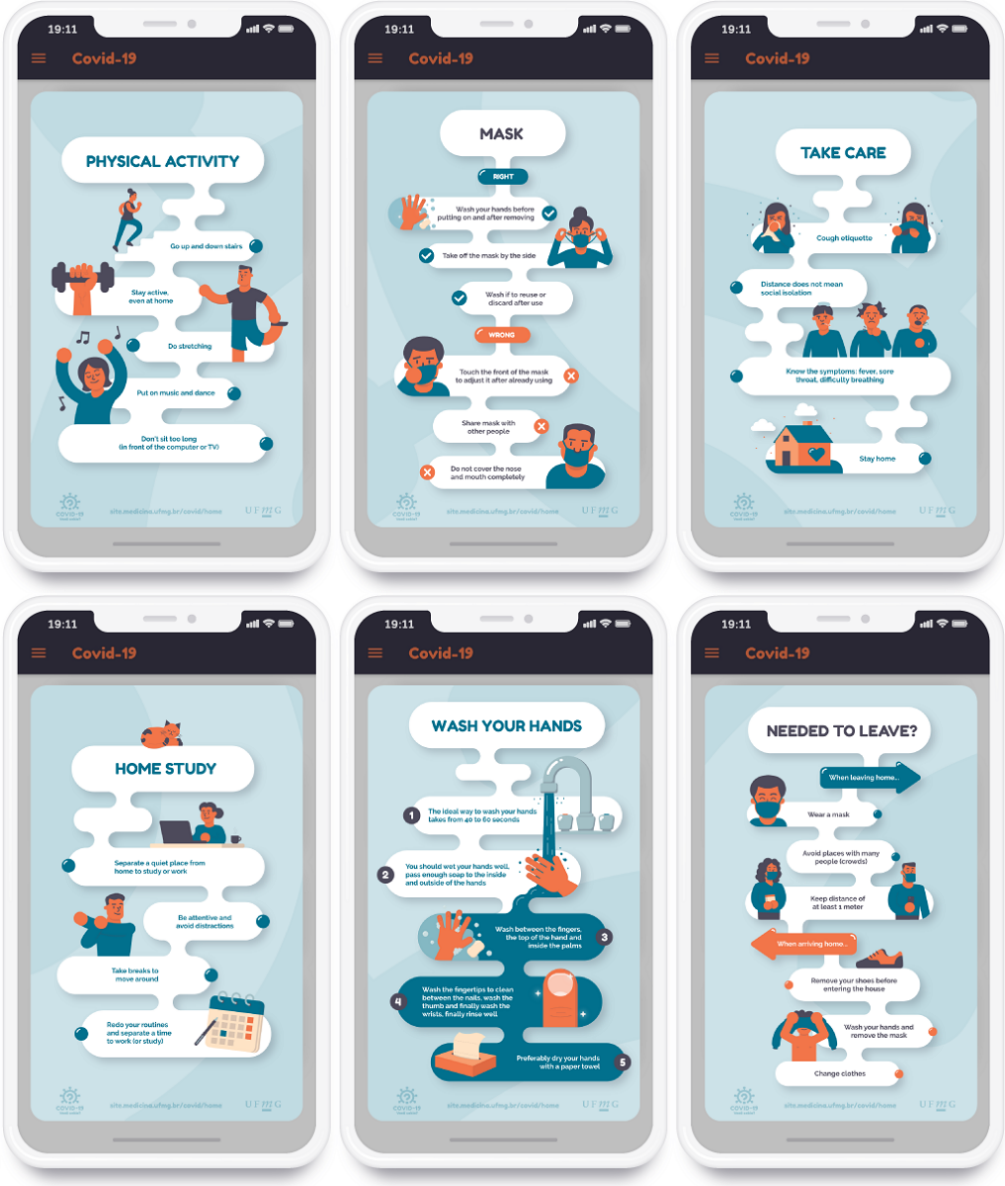
Infographics providing complementary information on the topics of the game.

The “Learn More” section presents infographics (Figure 6) with tips on how to proceed in situations related to each topic. Tips are provided for practicing physical activity, using masks, actions to take when leaving home, organizing work at home, hand washing care, and other personal care.

### Evaluation

**Figure 7:**
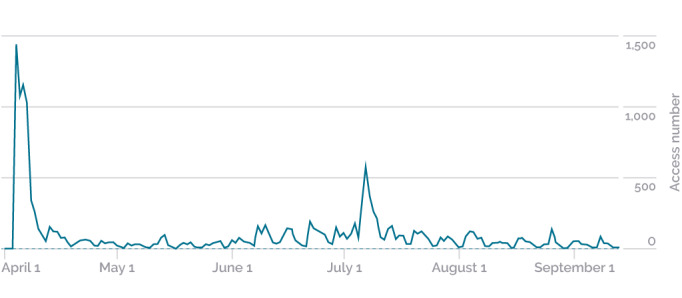
Distribution of game access between April and September 2020. Data are adapted from Google Analytics.

This topic shows the data extracted from the reports generated by Google Analytics. Between April 1 and September 13, the game was accessed 17,571 times (Figure 7). In this period, the primary type of device used to access the game was smartphones (79.8%), followed by computers (19.2%) and tablets (1.0%). The average duration of the game use time in the analyzed period was 3 minutes and 34 seconds.

**Figure 8:**
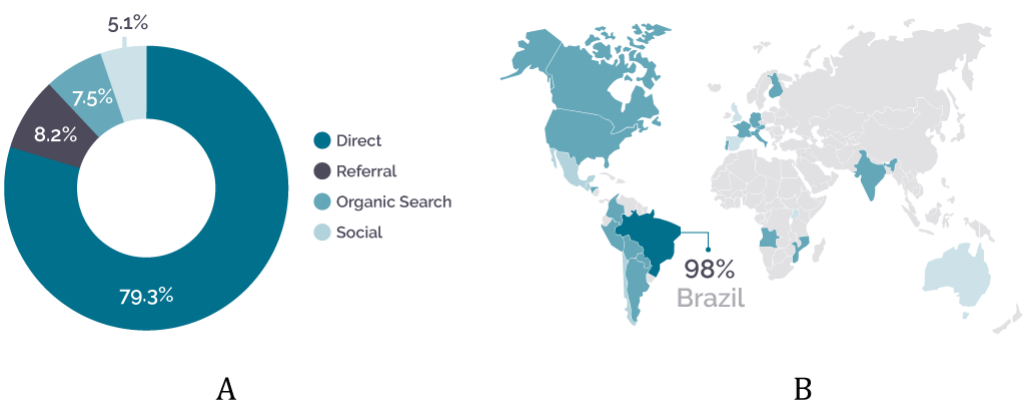
Distribution of game users by access channel (A) and by country (B) between April and September 2020. Adapted from Google Analytics.

Figure 8 contains two graphs showing how the users access the game website and where they access it from. Most of the access types are direct access (79.3%), which includes situations in which the user types the address directly into the browser, accesses links saved in favorites or links in PDF files, and in some cases accesses links in emails (Figure 8A). Then, there is access through references (8.2%), which includes access via links on other sites, such as news reports and articles. Moreover, 7.5% of users arrived at the site by searching for keywords related to the game on searching sites, and 5.1% of users accessed it through links on social networks. Most users were from Brazil (98%). This period of analysis precedes the publication date of the multilanguage version (Figure 8B).

**Figure 9:**
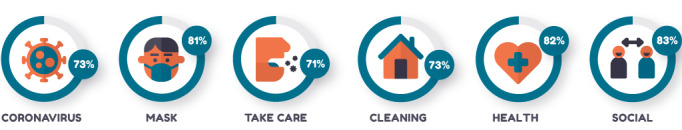
Correct answer rate for each game topic between July 20 and September 13, 2020.

Adjustments to the software and the Google Analytics settings available from July 20, 2020, also enabled us to extract the users' correct and incorrect answer rates from the reports by question, both individually and grouped by topic. The correct answer rates for the topics varied between 69% and 89%, as shown in Figure 9.

The error rates were grouped by topic and analyzed weekly (Table 1). A negative trend was observed only for the topic “Mask” (*r*=–.83), with a significant correlation (*P*=.01). The results for the other topics were not significant.

**Table 1:** Error rates by topic per week (July 20 to September 13, 2020).

Topic	Error rate per week (%)								*r^a^*	*P^b^*
	1	2	3	4	5	6	7	8		
Coronavirus	27.2	24.5	26.2	27.4	23.5	27.0	25.5	26.3	–.07	.87
Mask	17.0	17.3	19.2	18.8	14.8	13.7	12.2	12.2	–.83	.01
Take Care	33.0	29.8	30.5	32.4	30.0	31.5	28.8	33.7	–.002	.99
Cleaning	25.1	24.4	25.0	27.5	21.9	26.4	28.6	24.8	.25	.52
Health	18.1	17.4	18.6	18.0	17.9	17.9	19.2	19.1	.62	.08
Social	13.5	9.1	10.0	16.7	7.9	11.7	10.4	9.7	–.24	.53

^a^Pearson correlation coefficient.

^b^Student *t* test.

**Figure 10:**
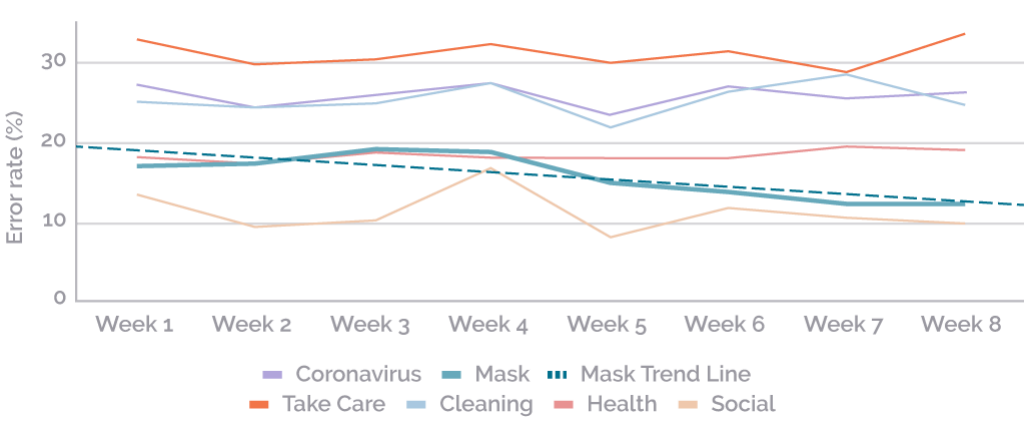
Error rates by topic grouped by week (July 20 to September 13, 2020). The linear trend for the Mask topic is significant (*r*=–.83).

The graph in Figure 10 shows the error rates grouped by topic over the weeks evaluated. The linear trend (*r*=–.83) for the significant topic (Mask) indicated a downward trend in the error rates.

### Publicizing Actions

**Figure 11:**
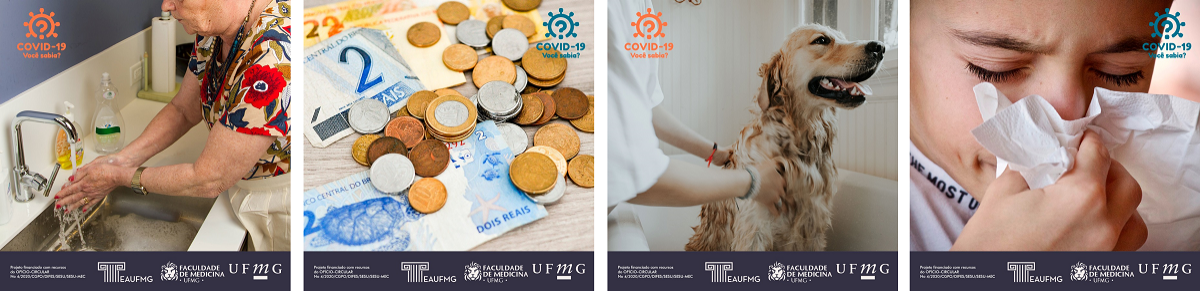
Examples of posts about the game on social networks.

Images and texts were produced by the design team related to the issues with the highest number of errors analyzed weekly (Figure 11). In this stage, images from the Unsplash [33] and Pexels [34] repositories that are offered free of charge by these platforms were searched for, selected, and used. From these images, graphic pieces were developed and published on the Facebook [25] and Instagram [26] accounts created for the project.

Other actions to publicize the serious game included sending informative emails to the Faculty of Medicine of UFMG newsletter mailing list as well as specific emails to teachers and coordinators of elementary and high schools. The game was also offered as an educational strategy for homeschooling. We contacted 2476 schools by email between April and May and 1020 additional schools in September.

The lowest bounce rates in the Google Analytics reports were observed for users who accessed the game through newspaper links, interviews, and links from Google Classroom (38%).

### Software Usability and Testing

The development team sought to meet the heuristic usability principles proposed by Nielsen and Molich [35]. We considered aspects such as offering simple dialogues, speaking the user's language, minimizing the user's memory overhead, maintaining consistent patterns of behavior and icons, offering continuous feedback to the user, providing demarcated exits with options to leave and return, providing shortcuts, avoiding error situations, offering clear error messages when necessary, providing an easy and intuitive interface, and offering help and clear documentation [36].

The serious game was subjected to empirical software development testing. White-box tests were performed to test game structures in specific parts of the development code for each component. Additionally, black-box tests were performed to validate the initially defined system requirements. Functional and nonfunctional items, such as performance, disclosure, acceptance, and release versions (alpha and beta), were evaluated. The observed inconsistencies were included in adjustments and incremental corrections in subsequent phases (sprints) of the development cycle.

Additionally, at least one visually impaired person played the game. His report was positive; he stated that he managed to use the game clearly with the aid of a specific reader for the visually impaired. He also reported the lack of information about the images related to the end of each topic. His feedback led to subsequent adjustments to improve the experience for visually impaired users, such as the inclusion of alternative text for these images.

## Discussion

### Principal Findings

The members of the multidisciplinary team, composed of physicians, designers, programmers, teachers, and students, were able to propose and develop solutions based on multiple aspects to implement the identified requirements, as recommended by Caserman and collaborators [15]. The weekly meetings were used as benchmarks to present the results of the week, evaluate the already implemented items, and propose new requirements for the development of an effective and attractive serious game.

The SCRUM development methodology proved to be efficient, enabling the division of the project into small increments; this methodology allowed for tests, rapid changes when necessary, and weekly deliveries to the end user. Functional software deliveries in a shorter period (1 week) generated greater customer satisfaction and provided a development environment with motivated individuals, as reported by Tobias and Spanier [37].

On January 30, 2020, the WHO declared that the outbreak of a disease caused by a new coronavirus, called COVID-19, constituted a Public Health Emergency of International Concern. This statement aimed to improve coordination, cooperation, and global solidarity to attempt to stop the spread of the new coronavirus. On March 11, 2020, the WHO characterized COVID-19 as a pandemic. Since the declaration of the outbreak and the characterization of the pandemic, the WHO has sought to inform the population about the health risks presented by COVID-19, considering that reliable information is as important as other protective measures. Well-informed people can make informed decisions and adopt positive behaviors to protect themselves and their families [38].

Much information about COVID-19 has been presented to the community; however, few initiatives are aimed at younger people. Thus, this audience was chosen because it has been more resistant to the recommendations of health authorities. Also, when teenagers are infected with SARS-CoV-2, they have few symptoms but can transmit the virus. We sought, through digital technology, to contribute to the rapid diffusion of information about SARS-CoV-2 and COVID-19, promoting changes in the attitude of the population [39].

Limited or insufficient health literacy has been associated with lower adoption of protective behaviors, such as vaccination, hand hygiene, and other self-care measures [40]. We avoided addressing specific issues in any country or region, avoiding major differences between the information provided by the WHO and the COVID-19 coping guidelines in each nation.

The use of PWA technology enabled the development of a hybrid app that combines the resources offered by browsers with the advantages of using smartphones, as in traditional apps [41]. Through this technology, the app is compatible with most browsers; thus, it can be used on different devices, such as computers, tablets, and smartphones [42].

Using the Web Storage technology offered by HTML5 enabled local storage of player data, such as their position in the game, correctly answered questions, and personal ranking, more securely than using cookies [22]. Development with Web Storage ensures that data will never be transferred to the server by the browser, as can happen with cookies, ensuring greater adherence to user data security policies.

Regarding the visual identity of the app, different factors were considered during development. The seriousness of the topic addressed required the promotion of accessibility, usability, and information. Thus, we considered the use of typography that offers good legibility, adequate contrast between colors, and icons and images that are easy to recognize [43,44]. Moreover, to guarantee access to simpler devices and reduce noise in the information, the visual identity was developed with the aim to achieve a lean and responsive design [45]. The other factor to consider is the fun aspect of games, in which more subjective issues such as attractiveness, entertainment, and aesthetics are addressed, as proposed by Caserman and collaborators [15]. To achieve this target, we used vibrant colors, flashy and stylized typography, illustrations, sounds, and graphic unity between the elements.

During the 6 months of game analysis, during which 17,500 users accessed the game, there were 2 months (April and July) in which television and newspaper reports about the game were added to the actions of sending informative emails with the college newsletter. The game was accessed mostly by smartphones (79.8%), which indicates that it is a good choice to prioritize resources and functionalities for this type of access over access by computers and tablets.

The weekly monitoring of error answer rates enabled us to improve the text of some questions and answers. Similarly, the weekly assessment of answers grouped by topic enabled the team to develop complementary actions with images and informative text for publication on social networks targeting the topics with the most mistakes that week.

The finding of statistical significance in reducing the trend of the error rate for the topic “Mask” raised some questions among the team. Because this study is not a controlled clinical trial, it is not possible to say that the population is more informed about the importance and use of masks; however, this issue stands out and suggests the need for further studies.

The lowest bounce rates in the Google Analytics reports were obtained for access from newspaper links, interviews, and Google Classroom. Considering that the bounce rate indicates when a user opens and then closes the website (without interacting with it), the lower bounce rates in these segments may indicate that focusing on news channels and teachers is the most assertive way to acquire new users of this serious game.

### Limitations

Because this is not a controlled study, the analysis permits limited inference that the results obtained are for a population of teenagers, which are the target population of this project. This is reinforced by the fact that the game does not have a user registry, is available on the internet, and is open to the community; therefore, the data analyzed in the current study do not enable inferences about who used the app.

Another limitation is that the sample used to assess the time series was seven weeks. Although statistical significance was found for one topic, evaluation for a longer period will be needed to state more safely that there is a decreasing error rate for that topic.

### Conclusions

This study managed to comply with the proposed objectives of developing a serious game and making it available to young people, providing reliable information on topics related to the prevention of COVID-19. Also, the multidisciplinary profile of the team was able to bring reflections of its paradigms to the project; therefore, the game achieves compliance with the technical, legal, functional, and attractiveness requirements expected for a serious game. Extrapolating the initial requirements, we performed promotion and dissemination actions and increased the accessibility of the game, making it multilingual and accessible to people with visual impairments. This publication can provide an example not only to other students and teachers but also for those with future interest in the application of good practices in the development of serious game apps. We hope that this game will continue to combat misinformation on the topic of COVID-19 and expand the population's engagement in preventive measures against the disease.

## Appendix

Multimedia Appendix 1 Privacy Policy.

## Abbreviations

API: application programming interface
CSS: cascading style sheets
GDPR: General Data Protection Regulation
HTML5: Hypertext Markup Language revision 5
PWA: progressive web app
UFMG: Universidade Federal de Minas Gerais
WHO: World Health Organization
