# Supplementary material for: A Mobile Serious Game About the Pandemic (COVID-19 - Did You Know?): Design and Evaluation Study
Source: JMIR Serious Games. 2020 Dec 22;8(4):e25226. doi: 10.2196/25226 (PMC7758085; doi:10.2196/25226)
Supplement: Multimedia Appendix 1 [file games_v8i4e25226_app1.pdf]

Privacy Policy

1. General information

This application is a contribution with information on ways to prevent the viral transmission of COVID-19 among people, especially among the young and adult-youth public and risk groups.

All the information contained was obtained through the recommendations of the World Health Organization (WHO), organized and validated by Physicians of the Federal University of Minas Gerais (UFMG). The developers (physicians and systems analysts) are willing to release updates in the contents according to significant updates of the WHO recommendations, so UFMG is not responsible for the release of outdated versions of the application containing contents that have become inadequate or that have proven to be ineffective over time. The protection instructions may change rapidly over time and for this, the user must keep the application updated with the new versions made available.

This application has no intention of providing specific guidelines for each location, so UFMG is not responsible for differences between the guidelines provided in the application based on WHO, with those recommended in the place where you live.

If you have any questions, please contact us at cins@medicina.ufmg.br. You can also write to us: Faculdade de Medicina da Universidade Federal de Minas Gerais, Avenida Alfredo Balena, 190, Sala 601, Santa Efigênica, Belo Horizonte, Minas Gerais, Brazil.

This Privacy Policy contains information regarding the way we treat, in whole or in part, in an automated manner or not, the personal data of users who access our application. Its purpose is to clarify those interested about the types of data that are collected, the reasons for the collection and how the user can update, manage or delete this information.

This Privacy Policy has been drawn up in accordance with Federal Law No. 12,965 of 23 April 2014 (Internet Civil Framework), Federal Law No. 13,709 of 14 August 2018 (Personal Data Protection Law) and EU Regulation No. 2016/679 of 27 April 2016 (General European Regulation on Personal Data Protection - PDRG).

This Privacy Policy may be updated as a result of any regulatory update, which is why the user is invited to consult this section periodically.

2. User Rights

The application is committed to comply with the standards set out in the PDRG, respecting the following principles:

- The user's personal data will be processed in a lawful, loyal and transparent way (lawfulness, loyalty and transparency);
- The user's personal data will be collected only for specified, explicit and legitimate purposes, and may not be further processed in a way incompatible with those purposes (purpose limitation);
- The user's personal data will be collected in an adequate, pertinent and limited way to the needs of the purpose for which they are processed (data minimization);
- The user's personal data will be accurate and updated whenever necessary, so that inaccurate data will be deleted or rectified when possible (accuracy);
- The user's personal data will be kept in a way that allows the identification of the data subjects only for the period necessary for the purposes for which they are processed (conservation limitation);
- The user's personal data will be treated in a safe way, protected from unauthorized or illicit treatment and against their loss, destruction or accidental damage, adopting the appropriate technical or organizational measures (integrity and confidentiality).

The user of the application has the following rights, conferred by the Personal Data Protection Act and the PDRG:

- Right of confirmation and access: it is the right of the user to obtain from the application the confirmation of whether or not personal data concerning him/her are being processed and, if so, the right to access his/her personal data;
- Right of rectification: it is the right of the user to obtain from the application, without undue delay, the rectification of inaccurate personal data concerning him/her;
- Right to delete data (right to forget): is the right of the user to have his data deleted from the application;
- Right to limitation of data processing: is the right of the user to limit the processing of his personal data, being able to obtain it when he disputes the accuracy of the data, when the processing is illicit, when the application no longer needs the data for the purposes proposed and when he has opposed the processing of the data and in case of processing of unnecessary data;
- Right of opposition: it is the right of the user to oppose at any time, for reasons related to his particular situation, the processing of personal data concerning him, and to oppose the use of his personal data to define a marketing profile (profiling);
- Right of data portability: it is the right of the user to receive the personal data concerning him/her that he/she has provided to the application, in a structured format, of current use and automatic reading, and the right to transmit such data to another application;
- The right not to be subjected to automated decisions: this is the right of the user not to be subject to any decision taken solely on the basis of automated processing, including profiling, which produces effects in his/her legal sphere or significantly affects him/her in a similar way.

The user may exercise his rights by means of written communication sent to the application with the subject "RGDP-", specifying:

- Full name or corporate name, CPF (Individual Taxpayer Registry) or CNPJ (National Registry of Legal Entities) number and e-mail address of the user and, if applicable, of his/her representative;
- Right you wish to exercise with the application;
- Date of request and signature of the user;
- Any document that can demonstrate or justify the exercise of your right.

The request should be sent to the following e-mail address: cins@medicina.ufmg.br, or by mail to: Faculdade de Medicina da Universidade Federal de Minas Gerais, Avenida Alfredo Balena, 190, Sala 601, Santa Efigênica, Belo Horizonte, Minas Gerais. The user will be informed in case of rectification or elimination of their data.

3. Duty not to provide third party data

While using the site, in order to safeguard and protect the rights of third parties, the user of the application should only provide their personal data, and not those of third parties.

4. Informações coletadas

A coleta de dados dos usuários se dará em conformidade com o disposto nesta Política de Privacidade e dependerá do consentimento do usuário, sendo este dispensável somente nas hipóteses previstas no art. 11, inciso II, da Lei de Proteção de Dados Pessoais.

4.1 Types of data collected

4.1.1 Sensitive data

No sensitive data will be collected from users, understood as those defined in articles 9 and 10 of the PDRG and in articles 11 and following of the Personal Data Protection Law. Thus, among others, the following data will not be collected:

- data revealing racial or ethnic origin, political opinions, religious or philosophical beliefs, or the user's union membership;
- genetic data;
- biometric data to uniquely identify a person;
- data concerning the health of the user;

- data relating to the user's sexual life or sexual orientation;
- data related to criminal convictions or violations or related security measures.

4.1.2 Collection of data not expressly provided for

Eventually, other types of data not expressly provided for in this Privacy Policy may be collected, provided that they are provided with the consent of the user, or that the collection is permitted or imposed by law.

There are situations where we request information about your opinion about the game and team suggestions, but you may refuse to respond. Outside of these situations, we prefer that you never share personal information with us.

Navigation Data

To improve your experience with this game, we use cookies and similar methods to recognize visitors and remember their score in the game.

Data obtained from third parties /h3>

We collect data about your use of the game from publicly available sources, service providers and partners who provide us with the data in accordance with the legislation. Some public information obtained by third parties, such as Google Analytics, may be used to improve your game experience (e.g.: game access device, computer, cell phone, smart TV and/or other access device; your geolocation; your IP address; type of operating system; connection information; device resolution; type of your browser; the pages you access within our game and hits or errors to the questions.

4.2 Legal basis for the processing of personal data

By using the application services, the user is consenting to this Privacy Policy.

The user has the right to withdraw his consent at any time, not compromising the lawfulness of the treatment of his personal data before the withdrawal. The withdrawal of consent may be made by e-mail: cins@medicina.ufmg.br, or by mail sent to the following address: Avenida Alfredo Balena, 190, Sala 601, Santa Efigênica, Belo Horizonte, Minas Gerais.

The consent of the relatively or absolutely incapable, especially of children under 16 (sixteen) years old, can only be made, respectively, if properly assisted or represented.

The processing of personal data without the user's consent will only be carried out on the basis of legitimate interest or for the hypotheses provided for by law, i.e., among others, the following:

- for the controller's compliance with a legal or regulatory obligation;
- for the carrying out of studies by a research agency, guaranteeing, whenever possible, the anonymization of personal data;
- when necessary for the execution of a contract or preliminary procedures related to a contract to which the user is a party, at the request of the data owner;
- for the regular exercise of rights in judicial, administrative or arbitral proceedings, the latter under the terms of Law No. 9307 of September 23, 1996 (Arbitration Law);
- for the protection of the life or the physical safety of the data subject or of third parties;
- for the protection of health, in procedures performed by health professionals or health entities;
- when necessary to meet the legitimate interests of the controller or third party, except in the case of prevailing fundamental rights and freedoms of the data owner that require the protection of personal data;
- for the protection of credit, including the provisions of the pertinent legislation.

4.3 Purposes of processing personal data

The personal data of the user collected by the application are intended to facilitate, expedite and fulfill the commitments established with the user and to enforce the requests made through the completion of forms.

The personal data may also be used for commercial purposes, to personalize the content offered to the user, as well as to subsidize the application to improve the quality and functioning of its services.

The treatment of personal data for purposes not foreseen in this Privacy Policy will only occur upon prior notice to the user, and in any case, the rights and obligations provided herein will remain applicable.

4.4 Period of conservation of personal data

The user's personal data will be kept for a maximum period of: zero days, unless the user requests its deletion before the end of this period.

The user's personal data may only be stored after the end of their processing in the following cases:

- for the controller's compliance with a legal or regulatory obligation;
- for study by a research agency, guaranteed, whenever possible, anonymization of personal data;
- for the transfer to a third party, provided that the data processing requirements established by law are respected;
- for the exclusive use of the controller, prohibited its access by third parties, and provided that the data are anonymized.

4.5 Recipients and transfer of personal data

The user's personal data will not be shared with third parties. Therefore, it will only be processed by this application.

5. Security in the processing of the user's personal data

The application undertakes to apply technical and organizational measures to protect personal data from unauthorized access and from situations of destruction, loss, alteration, communication or dissemination of such data.

To ensure security, solutions will be adopted that take into account: the appropriate techniques; the costs of implementation; the nature, scope, context and purposes of processing; and the risks to the rights and freedoms of the user.

However, the application disclaims liability for the exclusive fault of a third party, such as in the event of an attack by hackers or crackers, or the exclusive fault of the user, such as in the event that he transfers his data to a third party. The application also undertakes to notify the user in an appropriate time in case of any kind of violation of the security of his personal data that may cause him a high risk for his personal rights and freedoms.

The violation of personal data is a security violation that causes, in an accidental or illicit way, the destruction, loss, alteration, unauthorized disclosure or access to personal data transmitted, stored or subject to any other type of processing.

Finally, the application undertakes to treat the personal data of the user with confidentiality, within the legal limits.

6. Complaint to a supervisory authority

Without prejudice to any other administrative or judicial remedy, all data subjects have the right to lodge a complaint with a control authority. The complaint may be made to the authority of the application's headquarters, the user's country of habitual residence, his/her place of work, or the place where the infringement was allegedly committed.

7. Of the changes

This Privacy Policy was last updated on: 08/05/2020.

The editor reserves the right to modify, at any time and without any prior notice, the application the present rules, especially to adapt them to the evolutions of the application COVID-19 Did you know? either by providing new features, or by suppressing or modifying those already existing.

Therefore, the user is invited to periodically check this page for updates.

By using the service after eventual modifications, the user demonstrates his agreement with the new standards. If you disagree with any of the modifications, you must immediately

interrupt your access to the application and present your reservation to the customer service, if you so wish.

8. Applicable law and jurisdiction

For the resolution of the controversies arising from this instrument, Brazilian law will be fully applied.

Any disputes shall be presented in the jurisdiction where the application's editor is located.
